# Association between short-term exposure to air pollution and COVID-19: a case-crossover cohort study

**DOI:** 10.1136/bmjresp-2025-003279

**Published:** 2026-08-17

**Authors:** Andreas Tornevi, Christer Janson, Andreas Palm, Magnus Svartengren, Juan Wang, Andrei Malinovschi, Josef D Järhult, Mathias Holm, Sven-Erik Dahlen, Lars Modig, Bertil Forsberg, Össur Ingi Emilsson

**Affiliations:** 1 Department of Epidemiology and Global Health, Umea University, Umeå, Sweden; 2 Department of Medical Sciences, Respiratory, allergy and sleep research, Uppsala University, Uppsala, Sweden; 3 Department of Medical Sciences, Occupational and Environmental Medicine, Uppsala University, Uppsala, Sweden; 4 Department of Medical Sciences, Clinical physiology, Uppsala University, Uppsala, Sweden; 5 Department of Medical Sciences, Zoonosis Science Center, Uppsala University, Uppsala, Sweden; 6 Department of Occupational and Environmental Medicine, School of Public Health and Community Medicine, Sahlgrenska Academy, University of Gothenburg, Gothenburg, Sweden; 7 Department of Respiratory Medicine and Allergy, Karolinska University Hospital, Stockholm, Sweden; 8 Clinical Lung and Allergy Research Unit, Department of Medicine Huddinge, Karolinska Institutet and University Hospital, Stockholm, Sweden; 9 Faculty of medicine, University of Iceland, Reykjavík, Island

**Keywords:** COVID-19, Viral infection

## Abstract

**Objective:**

To investigate associations between short-term fluctuations in urban background air pollution and the incidence of COVID-19 across three Swedish cities using detailed individual-level data.

**Methods:**

A case-crossover analysis was conducted using individual-level data from a prospective cohort of adults across three Swedish cities (Stockholm, Gothenburg and Uppsala), including 3999 confirmed COVID-19 cases. Subjects were classified as cases on the date of their first positive PCR test, which occurred between 7 March 2020 and 26 January 2022. Exposure data for daily levels of PM2.5, PM10 and NO_2_ were obtained from urban background monitoring stations. Lag-specific and cumulative associations were examined using distributed lag models and distributed lag non-linear models, adjusting for meteorological variables.

**Results:**

A significant association was observed between elevated PM2.5 levels and testing positive for COVID-19 on the same day (lag 0), with an OR of 1.084 (95% CI 1.011 to 1.162) per IQR (3.3 µg/m³) increase. Similar lag 0 effects were found across the three cities. For PM10, the most consistent associations were observed at lag 3. For NO_2_, significant effects at lag 0 emerged only after excluding the final COVID-19 wave. Subgroup analyses showed similar effects among individuals with pre-existing respiratory conditions, though results were less consistent.

**Conclusions:**

Our findings suggest that short-term fluctuations in air pollution, particularly for PM2.5, are associated with the timing of confirmed COVID-19 cases. A possible explanation for the short lag association is that air pollution exacerbates symptoms, prompting individuals to seek testing, rather than directly facilitating new infections.

**WHAT IS ALREADY KNOWN ON THIS TOPIC**

- Short-term exposure to air pollution has been associated with increased COVID-19 incidence across multiple countries, typically using population-level time-series data.

**WHAT THIS STUDY ADDS**

- In this prospective cohort with individual-level PCR-confirmed COVID-19 diagnoses, same-day associations with PM2.5 were found, suggesting symptom exacerbation in already-infected individuals as a possible mechanism.

**HOW THIS STUDY MIGHT AFFECT RESEARCH, PRACTICE OR POLICY**

- These findings suggest that improving air quality may reduce symptom exacerbation in individuals with respiratory viral infections, representing potential public health benefits beyond chronic diseases.

## Introduction

Air pollution has long been recognised as a significant public health concern, contributing to approximately 7 million premature deaths annually.^1^ Both short- and long-term exposure to air pollution are associated with a broad range of respiratory and cardiovascular diseases.^2^ Fine particulate matter, particles with a diameter of up to 2.5 µm (PM2.5), also including very small particles generated by combustion, can be particularly harmful as they can penetrate deep into the lungs, enter the bloodstream and cross the blood-brain barrier, causing various adverse health effects including neural damage.^3 4^

In the context of airway infections such as coronavirus disease 2019 (COVID-19), caused by severe acute respiratory syndrome coronavirus 2 (SARS-CoV-2), there is a growing body of evidence that short-term fluctuations in air pollution play a role in the transmission and severity of the disease.^5^ This potential link could be due to several factors: air pollutants may act as carriers for the virus, prolonging the time for the virus to remain airborne and thereby increasing the risk of viral transmission; they may impair the immune system, making individuals more susceptible to infection; and they may exacerbate the severity of respiratory illnesses.^6 7^

A relationship between air pollution and increased risk of viral infections has been reported prior to the COVID-19 pandemic. An article from 2003 reported a link between an air pollution index in China and the severity of SARS outcomes.^8^ Another Chinese study from 2014 reported associations between PM2.5 and the number of human influenza cases.^9^ A study from Australia observed an elevated risk of paediatric influenza with increased daily levels of ambient PM10 (particles with a diameter of up to 10 µm) and ozone.^10^ However, a limitation of these studies is their reliance on data derived from healthcare settings, which likely under-represents the true spread of infection in the community by focusing primarily on severe cases detected through hospital admissions, while excluding undiagnosed or milder cases.

The COVID-19 pandemic, with thorough surveillance of confirmed cases, provides a unique opportunity to gain more insight into this topic. Studies have reported short-term associations between air pollutants and COVID-19 incidence in Asia,^11 12^ Europe^13 14^ and in North America.^15 16^ A systematic review from 2024 estimated a 5% increase in COVID-19 mortality for every 10 µg/m³ increment in both PM2.5 and PM10.^17^

The reported associations between air pollution exposure and increased COVID-19 incidence often have a short lag period (the delay between exposure and time of event), shorter than the incubation time for COVID-19, which has a mean value of around 5 days.^18^ This may suggest that air pollutants increase symptoms of an already manifested infection, rather than increasing the risk of becoming infected. In a study on young adults in Stockholm, Sweden, increased PCR-verified cases of COVID-19 were seen among young adults (born between 1994 and 1996) typically 2 days (lag 2) after increased exposure to PM2.5 and PM10.^19^ In a study from Spain, increased hospitalisations due to COVID-19 were observed on the same day (lag 0) as increased levels of nitrogen dioxide (NO_2_), PM10 and PM2.5.^14^

This study focused on the COVID-19 pandemic in Sweden from March 2020 to January 2022, aiming to examine fluctuations in ambient concentrations of PM2.5, PM10 and NO_2_ and their potential short-term association with the daily number of COVID-19 cases. The research used two general population study cohorts: Swedish participants from the Respiratory Health in Northern Europe (RHINE) study and the Global Allergy and Asthma European Network (GA2LEN) study. These cohorts also enabled analyses on susceptible groups, that is, individuals with documented respiratory diseases such as asthma and chronic obstructive pulmonary disease (COPD). Individuals with respiratory diagnoses have been shown to be particularly vulnerable to air pollution, and also severe COVID-19 outcomes.^20 21^

## Methods

### Individual data

To investigate associations between air pollution and the incidence of COVID-19, a population-based cohort study was constructed by combining data from the RHINE study^22^ and the GA2LEN study.^23^ The combined cohort is referred to throughout this manuscript as the REGAL cohort, an acronym derived from the names of the two source studies (RHINE, GA2LEN). These studies provide a rich dataset of participants from Sweden, originally residents in four Swedish cities: Stockholm, Gothenburg, Uppsala and Umeå. The RHINE study originally focuses on respiratory health across several Northern European countries, while the GA2LEN study is a large-scale network investigating allergy and asthma across Europe. These cohorts have, for example, information on education level and smoking habits (questionnaires from 2008 and 2010) and individuals’ height and weight. Data on age, sex and municipality of residence were collected from the national population register (Statistics Sweden). We linked the Swedish participant data with several national health registers, including the Patient Register, Cause of Death Register, Prescribed Drug Register and the national surveillance system for reportable infectious diseases (SmiNet), to obtain comprehensive health information. In total, the REGAL cohort included 36 798 individuals.

For this study, individuals with home addresses registered in Greater Stockholm, Gothenburg and Uppsala in late 2020 were included, as these cities have monitoring stations providing daily data on urban background levels of PM2.5, PM10 and NO_2_. A second inclusion criterion was having a positive PCR test for COVID-19 registered in SmiNet. Online supplemental figure S1 details the number of subjects included in the study.

The date of verified infection (case date) was defined as the date of testing. For participants with more than one positive test result, only the first was included in this study. Positive COVID-19 tests were recorded from 7 March 2020 to 26 January 2022.

### Exposure data

Daily mean values of ambient PM2.5, PM10, NO_2_ and meteorological data (temperature and relative humidity (RH)) for each city were sourced from the Swedish Meteorological and Hydrological Institute. Air pollution data were collected from one stationary measuring station for each city, representing urban background exposure. For Stockholm, daily levels of air pollution data were collected from a measuring station at Torkel Knutsongatan (rooftop, 20 m above street level). For Uppsala, a station located at Dragarbrunnsgatan (rooftop, height: 22 m) was used, and for Gothenburg, the station “Femman” (Spannmålsgatan) was used (rooftop, height: 30 m). Readings of daily mean values of temperature and RH were achieved from the stations Observatoriekullen (Stockholm), Landvetter Airport (Gothenburg) and Uppsala University. Air pollution data variables had missing values to various extents. In online supplemental figures S2–S4, air pollution data and daily frequency of COVID-19 cases for each city are illustrated.

### Susceptible group

In this study, individuals were classified as part of a susceptible group if they had a documented diagnosis of asthma or COPD or if they had documented use of inhaler medication. Asthma and COPD were defined based on diagnoses recorded in the Swedish Patient Register at any time before 2020, using International Classification of Diseases, 10th Revision codes J45–J46 for asthma and J44 for COPD. Participants were classified as using inhaler medication if they had two or more dispensed withdrawals from a pharmacy of any inhaler (ATC code R03) in 2019, the year preceding the pandemic.

### Statistical analysis

A time-stratified case-crossover study design with conditional logistic regression was used to estimate associations between air pollution and the incidence of positive PCR tests for COVID-19. Only individuals with positive COVID-19 tests in the REGAL cohort were included in analyses. In the case-crossover design, each case serves as their own control, thereby controlling for any time-invariant confounders (such as genetics, smoking, socioeconomics and chronic comorbidities) that remain constant over the short study period. This design was specifically chosen to focus on the acute triggering effects of air pollution while minimising confounding individual characteristics.

As delayed effects between air pollution and positive COVID-19 tests could be assumed, distributed lag models (DLM) and distributed non-linear lag models (DLNM) were applied.^24^ Each air pollutant (PM2.5, PM10 and NO_2_) was analysed in separate regression models (single pollutant models), and each model controlled for temperature and RH. The case-crossover design implies that each case date is compared with a selected control date/dates, and by choosing control days close in time with date of event (case date), trends are assumed to be controlled for. For each case, 1 week prior and 1 week after the case date were chosen for control days, thus also adjusting for any possible day-of-week trends. Additionally, a lag period of up to 2 weeks (0–13 days) was considered in the analyses. A lag period of up to 2 weeks (0–13 days) was chosen as a biologically reasonable time window to capture the incubation period, symptom onset and the subsequent delay in seeking a PCR test. This 14-day window implies, however, that for each case to be included in the regression model, using both control periods, no missing values are allowed in exposure variables 1 week ahead of the case date and 3 weeks prior to the case date. For this reason, sporadic missing values for one or two consecutive days in exposure variables were imputed and linearly interpolated. Longer periods of gaps in exposure data were left missing and thus excluded from analyses. Cases were only included if data allowed for two complete control periods. As there were different dates of missing values among air pollution variables (PM2.5, PM10 and NO_2_), the total number of cases included in the single pollutant models differs. Moreover, city-specific models were fitted independently to account for differences in pollutant baseline concentrations and meteorological conditions across cities, and results were then pooled across cities using a two-stage random-effects approach. Air pollution exposures were fitted with linear terms, and associations across lags were examined with different designs and lag periods. Initially, the cases which were recognised to be included (no missing data present) for a lag period of 0–13 days were identified. Then results from DLMs with different lag periods, from zero lags (a lag 0 model) up to 0–13 lags, were evaluated in terms of patterns of single lag effects and cumulative effects across lags. The full lag models (0–13 lags) were also fitted with penalised cubic regression splines across lags, with a maximum of 9 degrees of freedom and knots at equal distances (DLNM models).

To summarise, the outcome variable (dependent variable) was a binary indicator of COVID-19 case status: whether the observation date was the individual’s first positive PCR test date (case day, coded 1) or a matched control day (coded 0). The primary exposure variables (independent variables) were the daily mean concentrations of PM2.5, PM10 and NO_2_, each analysed in separate single-pollutant models. Temperature and RH were included as covariates in all models using the same distributed lag structure as the main exposure variable.

Cases with a historical record of respiratory diseases (susceptible group) were analysed in a complementary analysis and in the same way as the total population.

For Sweden (and the REGAL cohort), positive tests increased in December 2021 with very high frequencies throughout February 2022. As a sensitivity analysis, and to investigate if this period (‘the last COVID-19 wave’) could be considered influential to the results, analyses were made excluding this period (from 15 December 2021 and forward).

ORs with corresponding 95% CIs were estimated per 1 µg/m³ increase in daily mean concentration for each air pollutant. This scale was chosen to facilitate comparisons across models, as both the lag window length and the exclusion of certain time periods (sensitivity analyses) determine which calendar days contribute exposure data to the model and therefore affect the exposure distribution and any chosen summary measure of variability. A selection of results is also presented per IQR increase to aid interpretation of the magnitude of associations.

The statistical analysis was performed in R, using the dlnm package for distributed lag non-linear models and the mvmeta package for meta-analyses, applying random-effects with restricted maximum likelihood estimation to pool results across cities. Estimated ORs were considered statistically significant if their 95% CI did not encompass 1.

### Patient and public involvement

Patients or the public were not involved in the design, conduct, reporting or dissemination of this research. This study used existing cohort and registry data, and no direct patient or public engagement was applicable.

## Results

The total number of cases counted was 3999, of which 1767 were women and 2232 men. There were 1449 cases in Stockholm, 1544 in Gothenburg and 1006 in Uppsala, with a median age of 50 years (range 28–87 years). Due to missing values in air pollution variables, a total of 3824 unique individuals were included in at least one regression model. Among them, 386 individuals were diagnosed with asthma and/or COPD and/or were users of inhaler medications.

Descriptive statistics of the included population characteristics are presented in table 1, and summary statistics of air pollution data used in analyses for each city are shown in online supplemental table S1.

**Table 1:** Characteristics of the COVID-19 cases

Characteristic	Total population (n=3824)	Susceptible group (n=386)	Stockholm (n=1449)	Uppsala (n=973)	Göteborg (n=1402)
Sex: women, n (%)	1693 (44.3%)	177 (45.9%)	635 (43.8%)	449 (46.1%)	609 (43.4%)
Age, median (range) (years)	50.0 (28–87)	50.0 (28–87)	46.0 (28–87)	53.0 (28–87)	51.0 (29–87)
BMI, median (range)(kg/m²)*	23.8 (15.8–68.8)	23.9 (16.6–41.0)	23.2 (16.1–65.0)	24.2 (15.8–68.8)	24.2 (16.7–57.8)
Education: elementary, n (%)*	511 (15.0%)	71 (20.3%)	169 (12.6%)	129 (15.3%)	213 (17.3%)
Education: secondary, n (%)*	1186 (34.7%)	120 (34.4%)	407 (30.4%)	324 (38.4%)	455 (36.9%)
Education: college/university, n (%)*	1719 (50.3%)	158 (45.3%)	763 (57.0%)	391 (46.3%)	565 (45.8%)
Smoking: never, n (%)*	2072 (59.2%)	178 (49.3%)	810 (60.4%)	517 (58.8%)	745 (58.3%)
Smoking: prior, n (%)*	933 (26.7%)	115 (31.9%)	337 (25.1%)	244 (27.8%)	352 (27.5%)
Smoking: current, n (%)*	493 (14.1%)	68 (18.8%)	194 (14.5%)	118 (13.4%)	181 (14.2%)
COPD diagnosis: yes, n (%)†	61 (1.6%)	61 (15.8%)	23 (1.6%)	14 (1.4%)	24 (1.7%)
Asthma diagnosis: yes, n (%)†	196 (5.1%)	196 (50.8%)	78 (5.4%)	54 (5.5%)	64 (4.6%)
Inhaler medication: yes, n (%)†	293 (7.7%)	293 (75.9%)	124 (8.6%)	73 (7.5%)	96 (6.8%)
Missing data					
Missing: BMI	n=353 (9.2%)	n=34 (8.8%)	n=106 (7.3%)	n=126 (12.9%)	n=121 (8.6%)
Missing: education	n=408 (10.7%)	n=37 (9.6%)	n=110 (7.6%)	n=129 (13.3%)	n=169 (12.1%)
Missing: smoking	n=326 (8.5%)	n=25 (6.5%)	n=108 (7.5%)	n=94 (9.7%)	n=124 (8.8%)

Susceptible group: individuals with a diagnosis of asthma or COPD and/or dispensed inhaler medication.

* Percentages calculated among individuals with non-missing values.

† Asthma and COPD diagnoses based on the Swedish Patient Register (ICD-10: J45–J46 and J44, respectively). Inhaler medication use based on the Swedish Prescribed Drug Register (ATC: R03).

BMI, body mass index; COPD, chronic obstructive pulmonary disease.

**Figure 1:**
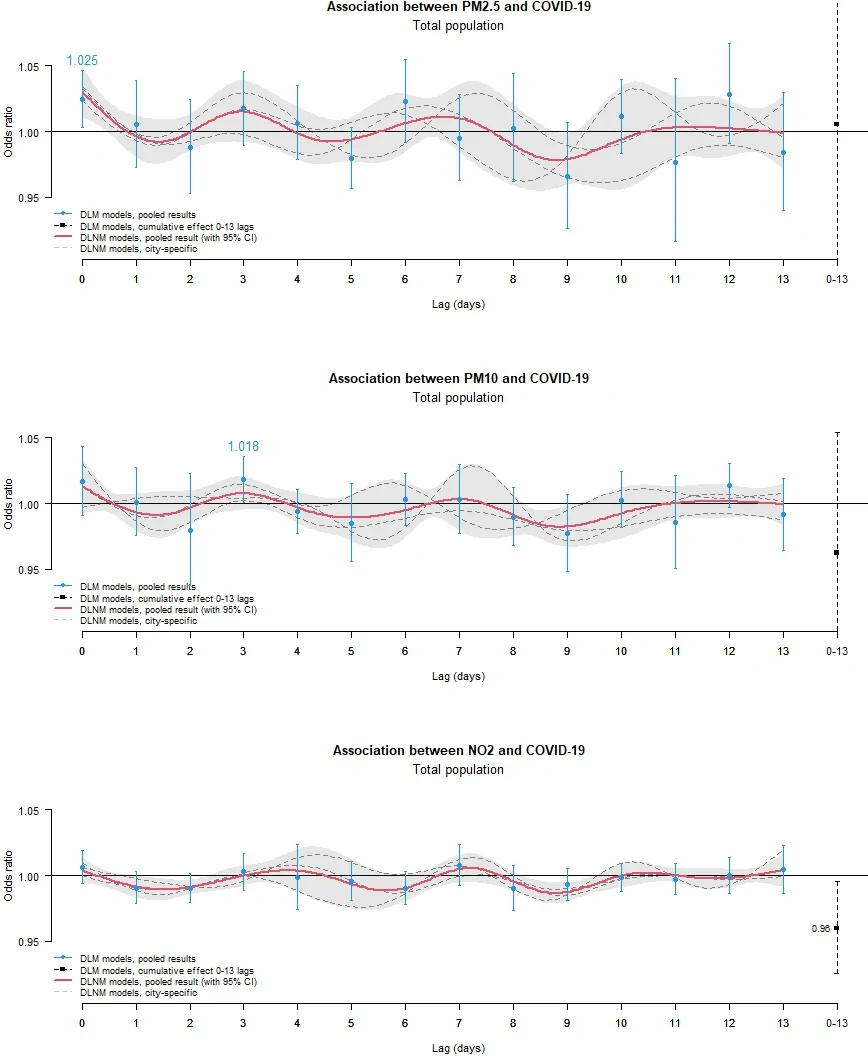
Total population analyses: lag-specific ORs (blue markers) and cumulative ORs (black squares) for COVID-19 per unit increase in exposure using pooled results from unconstrained lag models (DLM) and distributed lag non-linear models (DLNM) with penalised splines in lag-space. DLNM results are shown as the red line (pooled results) and grey dotted lines (city-specific associations). Error bars and grey shading indicate 95% CI. The top panel shows results for PM2.5 (n cases=3223), the middle panel for PM10 (n cases=3015) and for NO_2_ exposure in the bottom panel (n cases=3807). DLM, distributed lag models; NO_2_, nitrogen dioxide; PM, particulate matter.

**Figure 2:**
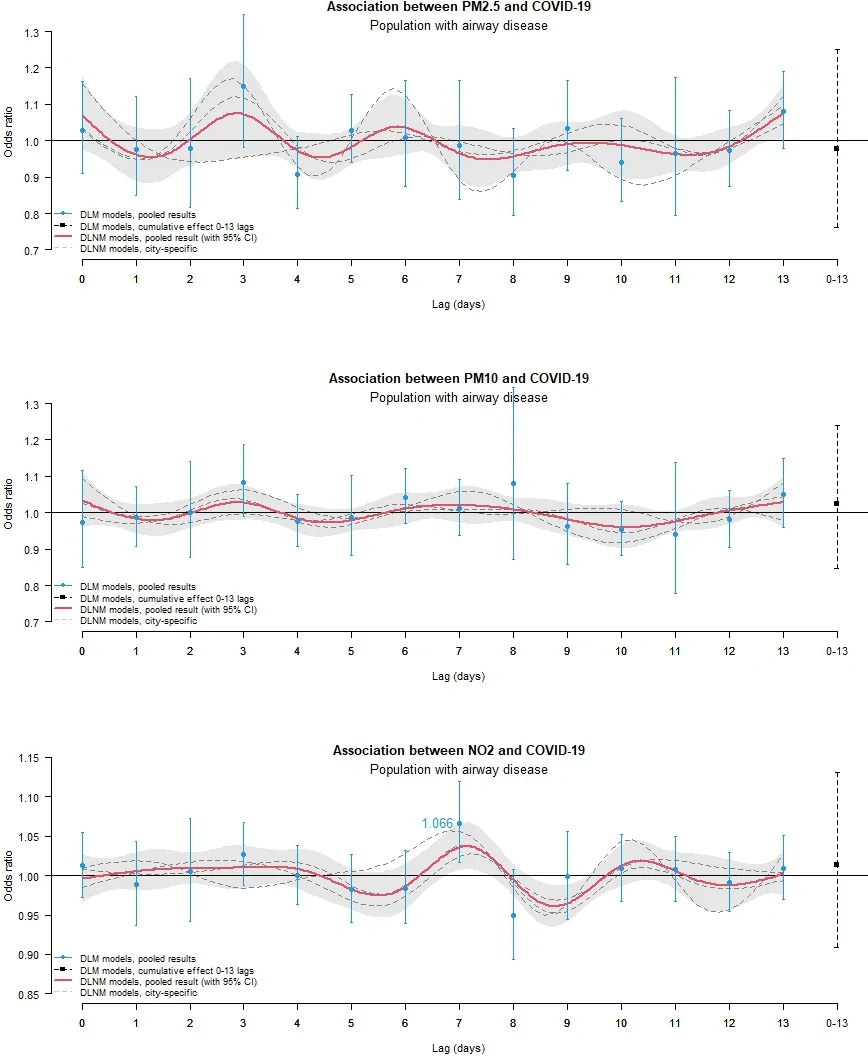
Susceptible group analyses (only including individuals with airway diseases): lag-specific ORs (blue markers) and cumulative ORs (black squares) for COVID-19 per unit increase in air pollution exposure using pooled results from unconstrained lag models (DLM) and distributed lag non-linear models (DLNM) with penalised splines in lag-space. DLNM results are shown as the red line (pooled results) and grey dotted lines (city-specific associations). Error bars and grey shading indicate 95% CI. The top panel shows results for PM2.5 (n cases=320), the middle panel for PM10 (n cases=310) and for NO_2_ exposure in the bottom panel (n cases=386). DLM, distributed lag models; NO_2_, nitrogen dioxide; PM, particulate matter.

**Figure 3:**
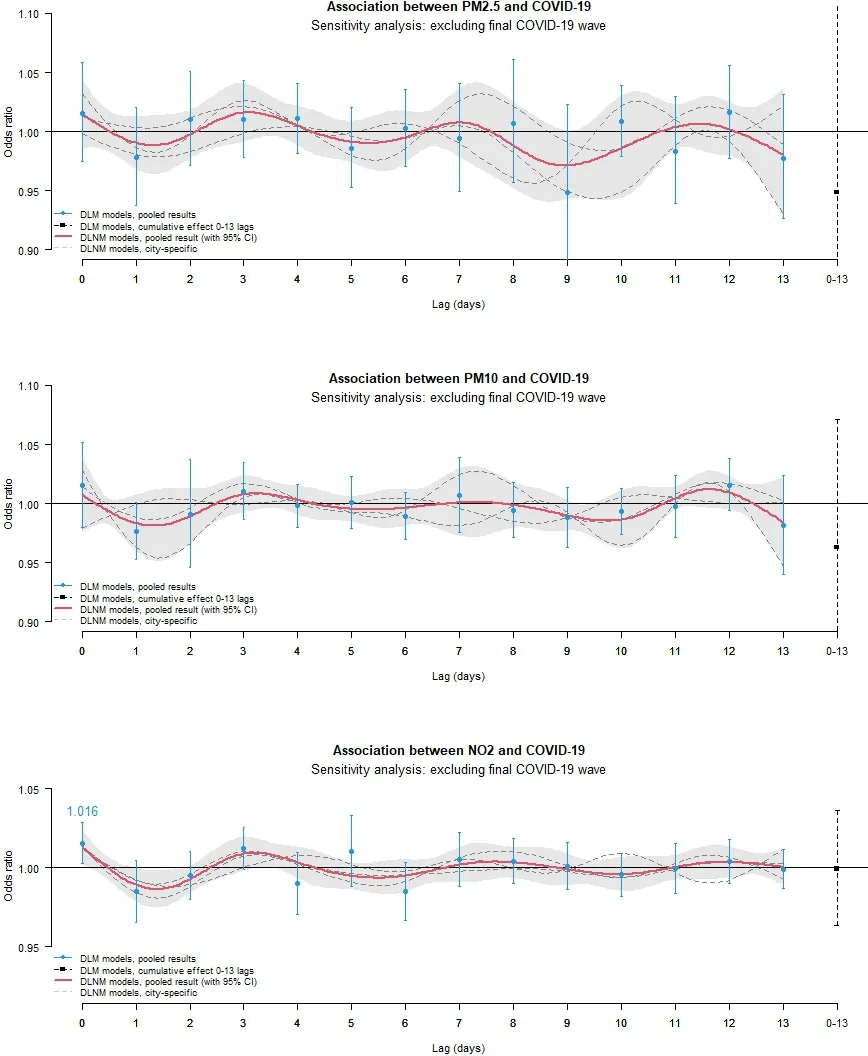
Sensitivity analyses (excluding all cases after 14 December 2021): lag-specific ORs (blue markers) and cumulative ORs (black squares) for COVID-19 per unit increase in exposure using pooled results from unconstrained lag models (DLM) and distributed lag non-linear models (DLNM) with penalised splines in lag-space. DLNM results are shown as the red line (pooled results) and grey dotted lines (city-specific associations). Error bars and grey shading indicate 95% CI. The top panel shows results for PM2.5 (n cases=1763), the middle panel for PM10 (n cases=1658) and for NO_2_ exposure in the bottom panel (n cases=2347). DLM, distributed lag models; NO_2_, nitrogen dioxide; PM, particulate matter.

In table 2, city-specific and pooled ORs at lag 0 and cumulative ORs for lag 0–6 and lag 0–13, by an IQR increase in each air pollutant, are displayed. Figure 1 illustrates ORs of becoming a case of a unit increase in each air pollutant across lags 0–13, including the total population. Figure 2 illustrates ORs of becoming a case of a unit increase in each air pollutant across lags 0–13, including only the population diagnosed with asthma/COPD and/or using inhaler medications (susceptible group). Figure 3 illustrates ORs of becoming a case of a unit increase in each air pollutant across lags 0–13, including the total population and omitting the last COVID-19 wave (sensitivity analyses). In online supplemental figures S5–S7, results from sensitivity analyses are shown exploring models with different lengths of lag windows for PM2.5. Similar results are shown in online supplemental figures S8–S10 for PM10 and in online supplemental figures S11–S13 for NO_2_.

**Table 2:** City-specific and pooled ORs with 95% CIs for the association between air pollutants and COVID-19, expressed per IQR increase in daily mean concentration

Pollutant	City	Increment (IQR)	OR lag 0	OR cumulative lag 0–6	OR cumulative lag 0–13
PM2.5	Stockholm	3.19	1.079 (0.981 to 1.187)	1.165 (1.003 to 1.352)	1.336 (1.067 to 1.672)
PM2.5	Uppsala	3.11	1.095 (0.957 to 1.253)	1.227 (1.011 to 1.489)	1.185 (0.891 to 1.576)
PM2.5	Gothenburg	3.56	1.074 (0.928 to 1.244)	1.062 (0.842 to 1.339)	0.610 (0.426 to 0.872)
PM2.5	Pooled results	3.29	1.084 (1.011 to 1.162)	1.152 (1.005 to 1.321)	1.018 (0.647 to 1.601)
PM10	Stockholm	6.01	0.976 (0.848 to 1.123)	0.850 (0.691 to 1.045)	0.823 (0.634 to 1.070)
PM10	Uppsala	5.79	1.114 (0.974 to 1.274)	1.230 (1.001 to 1.511)	1.236 (0.914 to 1.670)
PM10	Gothenburg	6.5	1.269 (1.032 to 1.560)	0.882 (0.636 to 1.224)	0.432 (0.265 to 0.706)
PM10	Pooled results	6.1	1.108 (0.947 to 1.296)	0.981 (0.754 to 1.276)	0.792 (0.456 to 1.376)
NO_2_	Stockholm	4.9	1.059 (0.977 to 1.148)	0.743 (0.623 to 0.886)	0.711 (0.547 to 0.924)
NO_2_	Uppsala	4.3	1.053 (0.931 to 1.192)	1.055 (0.847 to 1.314)	1.091 (0.784 to 1.518)
NO_2_	Gothenburg	8.47	0.990 (0.891 to 1.100)	0.811 (0.663 to 0.991)	0.680 (0.499 to 0.927)
NO_2_	Pooled results	5.89	1.038 (0.964 to 1.117)	0.862 (0.671 to 1.107)	0.786 (0.633 to 0.975)

Results are based on distributed lag models (DLM).

NO_2_, nitrogen dioxide; PM, particulate matter.

### COVID-19 and particulate matter (PM2.5)

For PM2.5, the number of cases included (after cleaning for missing values) was 3223 (1337 cases in Stockholm, 1000 cases in Gothenburg and 886 cases in Uppsala). PM2.5 levels and testing positive for COVID-19 were associated most clearly on the day of exposure (lag 0). In figure 1 (top panel), the pooled results from city-specific DLM and DLNM models using 0–13 lags with PM2.5 as the exposure variable are shown, where a one-unit increase in PM2.5 (µg/m³) was associated with a 2.4% increase in the odds of becoming a case at lag 0. This corresponds to an OR of 1.084 per IQR increase in PM2.5 (mean IQR=3.3 µg/m³; table 2). The lag 0 association between PM2.5 and COVID-19 remained stable independently of the number of lags included in the regression models. Specifically, for a lag 0 model (no lagged associations estimated in the model), one unit increase in PM2.5 (µg/m³) was associated with a 2.5% increase in the odds of becoming a case. (OR 1.025; 95% CI 1.008 to 1.042) (online supplemental figure S5). All models up to lag 0–13 showed significant associations at lag 0, with ORs spanning 1.021–1.031. Cumulative effects over lags were significant in all models up to lag 0–7, with ORs spanning 1.025 (lag 0–1 model) up to 1.043 (lag 0–6 model) (online supplemental figure S5). In models analysing associations longer than 7 lags, the cumulative effects became insignificant in DLM models.

For the susceptible group (individuals with pre-existing respiratory conditions as described above, n=320 for PM2.5 analysis), a significant association was observed at lag 0 with an OR of 1.052 (95% CI 1.007 to 1.099), in a lag 0 model (online supplemental figure S6). Longer lag models also showed effects at lag 3, but did not show significance in all models. Full-length lag models (0–13 lags) did not show any significant associations between PM2.5 and risk of COVID-19 for this susceptible group (figure 2).

### COVID-19 and particulate matter (PM10)

For PM10, 3015 cases were included for analysis (1123 cases in Stockholm, 1006 cases in Gothenburg and 886 cases in Uppsala). In a lag 0 model, an OR of 1.014 (95% CI 1.000 to 1.029) for each unit increase in PM10 was observed, but the lag 0 effect became non-significant in longer lag models. Longer lag models (0–4 up to 0–13) showed a significant association at lag 3 with ORs spanning 1.014–1.019 depending on the number of lags included (online supplemental figure S8). A penalised spline across lags 0–13 also showed a significant increase around lag 3 and a negative association around lag 9, which was not recognised in DLM models (figure 1, middle panel).

For the susceptible group (n=310 for PM10), no significant lag-specific association or cumulative effects were observed (figure 2 and online supplemental figure S9).

### COVID-19 and nitrogen dioxide (NO_2_)

For NO_2_, 3807 cases were identified to be included for analysis (1443 cases in Stockholm, 1391 cases in Gothenburg and 973 cases in Uppsala). Negative lag-specific associations were observed in some models, and in longer lag models (> 8 lags), negative cumulative effects were observed (figure 1, bottom panel, online supplemental figure S11).

For the susceptible group (n=386), an effect at lag 7 of NO_2_ exposure was observed, followed by decreased risk at lag 8 in the full-length lag models (figure 2, bottom panel). No cumulative effects were noted for NO_2_ (online supplemental figure S12).

### Sensitivity analyses

In the sensitivity analysis for PM2.5, excluding the last COVID-19 wave (limiting the analysis to 1763 cases), lag 0 showed associations with COVID-19 similarly to when studying the full period but did not reach significance in all models (online supplemental figure S7). Collectively, PM2.5 demonstrated an effect at lag 0 in all models with various lag lengths, and when excluding the last COVID-19 wave (reducing cases by approximately 45%), the models indicated similar ORs.

For PM10, excluding the last COVID-19 wave (limiting the number of cases to 1,658), the association observed at lag 3 became insignificant, and some models also indicated negative associations at lag 1 (online supplemental figure S10).

For NO_2_ and excluding the last COVID-19 wave, lag 0 became significantly positive across all models (total population, n cases=2347) (figure 3 and online supplemental figure S13). ORs at lag 0 spanned between 1.011 (lag 0 model) and 1.015 (95% CI 1.002 to 1.029) in a 0–13 lag model, per unit increase in NO_2_ (online supplemental figure S13). Thereby, the last COVID-19 wave (with many cases registered during a short period) was shown to impact the risk estimates for NO_2_, since excluding this period revealed a lag 0 effect for NO_2_.

## Discussion

This large general population-based study provides evidence of a significant association between short-term exposure to ambient air pollution and the timing of testing positive for COVID-19 in Sweden. Our comprehensive analysis therefore confirms and extends previous research suggesting that air pollutants may exacerbate viral infections and symptoms, contributing to an increased number of COVID-19 cases.^14 16^ For specific lags, the clearest association was observed on the day of exposure (lag 0), where a 2.5% increase in the odds of testing positive for COVID-19 was noted per unit increase in PM2.5. This immediate effect was consistent across all three studied cities, suggesting that short-term fluctuations in air pollution might increase susceptibility to existing infections or promote symptom development, prompting testing and diagnosis.

For individuals with pre-existing respiratory conditions such as asthma or COPD, a significant association with PM2.5 was observed at lag 0 and lag 3 in some models and at lag 7 for NO_2_. However, these effects were less consistent, indicating that while this group may be particularly vulnerable to air pollution, the findings require cautious interpretation due to the smaller sample size (~10% of cases). The susceptible group was defined conservatively, based on verified diagnoses of asthma or COPD in the Swedish Patient Register and/or dispensed inhaler medications from the Prescribed Drug Register. While this approach ensures objective ascertainment of clinically significant, treatment-requiring airway disease, it likely underestimates the true prevalence of susceptible individuals in the cohort, as those with mild or undiagnosed conditions would not be captured. To the best of our knowledge, no other study has investigated the association between air pollution and COVID-19 incidence specifically focusing on individuals with pre-existing respiratory conditions. However, Uruma *et al* demonstrated in a meta-analysis that individuals with asthma or COPD are over-represented among COVID-19 cases, highlighting the heightened vulnerability of this group to both infection and severe outcomes.^25^

For PM10, in addition to an immediate effect at lag 0 (in a lag 0 model), significant associations were most consistently observed at lag 3 across different models, indicating a pattern similar to the estimated effects of PM2.5. Generally, PM2.5 and PM10 are highly correlated, making it challenging to disentangle their individual effects, even though their physical and chemical properties can differ and also vary by location.^26^ This overlap complicates the precise attribution of health impacts to PM2.5 or PM10 alone, but the findings in this study suggest a comparatively stronger risk associated with PM2.5.

A notable increase in COVID-19 cases occurred at the end of 2021, coinciding with government recommendations to work from home and stricter social distancing rules implemented from December 23. When excluding this period from the analyses, a lag 0 effect also became visible for NO_2_, which strengthens a hypothesis that air pollution might heighten symptoms and thereby encourage testing. The estimated effect at lag 0 of NO_2_ exposure pointed to an OR of 1.011–1.016 (depending on the model) per unit increase. This is in line with what a world-wide meta-analysis reported, covering 68 estimates from both low-income and high-income countries, where the pooled short-term association of NO_2_ was 1.014 (95% CI 1.011 to 1.016) per unit increase.^27^

The meta-analysis by Zang and coworkers reported a lower impact on PM2.5 and PM10 than this present study, with risk estimates of 1.003 for PM2.5 and 1.005 for PM10 of a one-unit increase.^27^ Our risk estimates for PM2.5 and PM10 are, though, approximately in line with a similar short-term assessment on young adults in Stockholm by Yu *et al*, which reported an increase of around 7% of COVID-19 cases of an IQR increase in PM2.5 and PM10 at lag 2.^19^ Corresponding estimates in this present study (by a IQR increase in air pollutants at lag 0) was 8% and 11% increased risk for PM2.5 and PM10, respectively. Yu *et al* also used a similar statistical design (DLNM case-crossover) as the present study but did employ a dispersion model for air pollutants targeting the included individuals’ home address. The reason for this discrepancy between Zang’s meta-analysis and the Swedish data is not clear. Possibly, changes in the generally low levels of air pollution in Sweden may have a greater impact on health than variations at higher levels of air pollution. This would indicate that even in areas of generally lower levels of air pollution, there is a significant health benefit of decreasing air pollution.

If air pollution primarily facilitated new SARS-CoV-2 infections, associations would be expected to emerge with a delay corresponding to the incubation period, on average approximately 5 days. The consistent lag 0 finding, rather than associations around lag 5, suggests that air pollution may not act solely as a facilitator of new infection. Instead, this pattern is more consistent with a mechanism whereby short-term increases in air pollution exacerbate respiratory inflammation and symptoms in individuals who are already infected but asymptomatic or mildly symptomatic, thereby prompting them to seek a PCR test. This interpretation is supported by the consistency of the lag 0 effect across all three cities, suggesting a genuine biological signal. We therefore interpret our findings primarily as indications that air pollution may accelerate the onset of symptoms and subsequent COVID-19 diagnosis, rather than exclusively increasing the underlying risk of infection. This biological signal is plausible, given that exposure to pollutants like NO_2_ and PM2.5 is known to induce oxidative stress and airway inflammation, potentially lowering the threshold for symptomatic expression of an ongoing viral infection. The stronger and more consistent associations observed for PM2.5 compared with PM10 are consistent with differences in particle size, chemical composition and respiratory deposition. PM2.5 consists predominantly of combustion-derived particles capable of penetrating deep into the alveoli, where they may trigger local and systemic inflammatory responses. PM10 additionally includes coarser, locally-generated particles such as road dust and tyre wear, deposited more proximally in the upper airways. Although the two fractions are correlated, making independent attribution difficult in single-pollutant models, the deeper penetration and higher biological potency of PM2.5 may explain why its association with respiratory outcomes is often more pronounced. Regarding the study period, it is important to note that Sweden did not implement a full national lockdown. However, voluntary social distancing and reduced mobility likely influenced local emission patterns during certain phases of the pandemic.

### Strengths and limitations

The strengths of the present study include its large cohort, spanning multiple cities with a long observation period and a case-crossover design that eliminates time-invariant confounders by allowing each case to serve as its own control. The statistical design, which uses control days close to the case date (1 week before and after), also minimises the impact of behavioural trends in seeking PCR tests, including day-of-week effects.

Several limitations should be considered when interpreting the findings of this study. Although the models controlled for key meteorological variables such as temperature and RH, the potential for confounding by other factors remains. Individual behaviours, including mask-wearing, social distancing and indoor air quality, as well as variations in COVID-19 testing strategies, were not accounted for in this study. These factors could influence both exposure levels and the likelihood of being diagnosed with COVID-19. As these behaviours evolved throughout the pandemic,^28^ we sought to minimise this risk by first analysing the entire study period and then excluding the last and largest COVID-19 wave, dominated by the Omicron variant, where behaviours likely differed. Furthermore, the patterns in the three cities were largely similar despite differences in testing strategies and wave periods, suggesting that this bias did not significantly affect our findings.

Another limitation is that exposure assessment was based on one urban background monitoring station per city. While this approach is commonly used in time-series and case-crossover studies to capture temporal variation in ambient pollution, individual exposure levels may differ due to local traffic conditions, workplace environments or indoor exposure. Because such measurement errors are likely unrelated to the timing of SARS-CoV-2 infection, any resulting bias would most likely be towards the null.

Finally, although PM2.5, PM10 and NO_2_ were analysed separately, there remains uncertainty around pollutant specificity. Interactions between different pollutants or the combined effects of multiple pollutants were not explored here. Therefore, it is unclear whether the associations observed are specific to each individual pollutant or reflect the cumulative effects of multiple environmental stressors.

## Conclusion

Our findings suggest that short-term fluctuations in air pollution, particularly PM2.5, are associated with the timing of confirmed cases of COVID-19. A possible explanation for the association at short lags is that air pollution exacerbates respiratory symptoms, prompting individuals to seek testing. The results should be interpreted with the limitations of an observational design in mind. These findings add to a growing body of evidence suggesting that air pollution may play a role in respiratory viral infections and highlight the potential public health relevance of air quality improvements for respiratory health more broadly.

## Supplementary material

10.1136/bmjresp-2025-003279 Supplementary file 1

## Data Availability

Data are not available upon reasonable request.
