# Supplementary material for: Association between short-term exposure to air pollution and COVID-19: a case-crossover cohort study
Source: BMJ Open Respir Res. 2026 Aug 17;13(1):e003279. doi: 10.1136/bmjresp-2025-003279 (PMC13504881; doi:10.1136/bmjresp-2025-003279)
Supplement: Supplementary file 1 [file bmjresp-13-1-s001.docx]

**Association between short-term exposure to air pollution and COVID-19: Supplementary material**

**Figure S1. Flow chart.**

**Table S1. Summary statistics of air pollution observations**

**Figure S2. Data for Stockholm.**

**Figure S3. Data for Gothenburg.**

**Figure S4. Data for Uppsala.**

**Figure S5. Sensitivity analyses: PM2.5, total population.**

**Figure S6. Sensitivity analyses: PM2.5, susceptible group.**

**Figure S7. Sensitivity analysis: PM2.5, excluding last COVID-19 wave.**

**Figure S8. Sensitivity analyses: PM10, total population.**

**Figure S9. Sensitivity analyses: PM10, susceptible group.**

**Figure S10. Sensitivity analysis: PM10, excluding last COVID-19 wave.**

**Figure S11. Sensitivity analyses: NO2, total population.**

**Figure S12. Sensitivity analyses: NO2, susceptible group.**

**Figure S13. Sensitivity analysis: NO2, excluding last COVID-19 wave.**

**Figure S1. Flow chart.** Number of individuals included/excluded for the study.

**Table S1.** Summary statistics of air pollution observations included in analysis (case dates and lags).

|  | Minimum | Q1 | Median | Q3 | Maximum | Mean | SD | n dates |
| --- | --- | --- | --- | --- | --- | --- | --- | --- |
| Stockholm |  |  |  |  |  |  |  |  |
| PM2.5 | 0.7 | 2.2 | 3.5 | 5.5 | 29.8 | 4.5 | 3.7 | 574 |
| PM10 | 0.2 | 5.9 | 8.3 | 12.2 | 87.4 | 9.9 | 7.0 | 588 |
| NO2 | 2.3 | 5.9 | 7.9 | 10.5 | 55.2 | 9.1 | 5.2 | 647 |
| Gothenburg |  |  |  |  |  |  |  |  |
| PM2.5 | 0.9 | 3.6 | 4.9 | 7.1 | 25.0 | 5.9 | 3.5 | 424 |
| PM10 | 0.3 | 7.4 | 10.0 | 13.9 | 74.7 | 11.1 | 6.2 | 438 |
| NO2 | 1.3 | 7.1 | 10.4 | 15.3 | 57.3 | 12.4 | 7.7 | 682 |
| Uppsala |  |  |  |  |  |  |  |  |
| PM2.5 | 0.6 | 2.1 | 3.3 | 5.4 | 26.5 | 4.2 | 3.2 | 606 |
| PM10 | 1.1 | 4.0 | 6.6 | 10.0 | 38.6 | 7.7 | 5.1 | 606 |
| NO2 | 0.8 | 3.4 | 5.0 | 7.2 | 34.8 | 5.9 | 4.1 | 641 |

**Figure S2**. **Data for Stockholm**. Daily air pollution observations in Stockholm for PM2.5 (top), PM10 (middle), and NO2 (bottom). Observed and interpolated pollutant concentrations are distinguished by color. Gray bars represent the daily number of COVID-19 cases included in the single-pollutant models after cleaning for missing observations in air pollution data.

**Figure S3. Data for Gothenburg.** Daily air pollution observations in Gothenburg for PM2.5 (top), PM10 (middle), and NO2 (bottom). Observed and interpolated pollutant concentrations are distinguished by color. Gray bars represent the daily number of COVID-19 cases included in the single-pollutant models after cleaning for missing observations in air pollution data.

**Figure S4. Data for Uppsala.** Daily air pollution observations in Uppsala for PM2.5 (top), PM10 (middle), and NO2 (bottom). Observed and interpolated pollutant concentrations are distinguished by color. Gray bars represent the daily number of COVID-19 cases included in the single-pollutant models after cleaning for missing observations in air pollution data.

**Figure S5. Sensitivity analyses: PM2.5, total population**. Lag-specific odds ratios (red markers) and cumulative odds ratios (black squares) for COVID-19 per unit increase in air pollution exposure for PM2.5 in unconstrained lag models with different lag length (total population, pooled results from 3 cities, n cases= 3,223). Bars represent 95% CI, and significant associations are annotated with the corresponding odds ratio estimates.

**Figure S6. Sensitivity analyses: PM2.5, susceptible group**. Lag-specific odds ratios (red bars) and cumulative odds ratios (black bars) for COVID-19 per unit increase in air pollution exposure for PM2.5 in unconstrained lag models with different lag length (susceptible group, pooled results from 3 cities, n cases=320). Bars represent 95% CI, and significant associations are annotated with the corresponding odds ratio estimates.

 **Figure S7.** **Sensitivity analysis: PM2.5, excluding last COVID-19 wave.** Lag-specific odds ratios (red bars) and cumulative odds ratios (black bars) for COVID-19 per unit increase in PM2.5 exposure in unconstrained lag models with varying lag lengths (pooled results from 3 cities, excluding data from the final COVID-19 wave, n cases= 1,763). Bars represent 95% CI, and significant associations are annotated with the corresponding odds ratio estimates.**Figure S8.** **Sensitivity analyses: PM10, total population.** Lag-specific odds ratios (red markers) and cumulative odds ratios (black squares) for COVID-19 per unit increase in air pollution exposure for PM10 in unconstrained lag models with different lag length (total population, pooled results from 3 cities, n cases= 3,015). Bars represent 95% CI, and significant associations are annotated with the corresponding odds ratio estimates.

.

**Figure S9.** **Sensitivity analyses: PM10, susceptible group.** Lag-specific odds ratios (red markers) and cumulative odds ratios (black squares) for COVID-19 per unit increase in air pollution exposure for PM10 in unconstrained lag models with different lag length (susceptible group, pooled results from 3 cities, n cases = 310). Bars represent 95% CI, and significant associations are annotated with the corresponding odds ratio estimates.

**Figure S10**. **Sensitivity analysis: PM10, excluding last COVID-19 wave.** Lag-specific odds ratios (red markers) and cumulative odds ratios (black squares) for COVID-19 per unit increase in PM10 exposure in unconstrained lag models with varying lag lengths (pooled results from 3 cities, excluding data from the final COVID-19 wave, n cases = 1,658). Bars represent 95% CI, and significant associations are annotated with the corresponding odds ratio estimates

**Figure S11**. **Sensitivity analyses: NO2, total population.** Lag-specific odds ratios (red markers) and cumulative odds ratios (black squares) for COVID-19 per unit increase in air pollution exposure for NO2 in unconstrained lag models with different lag length (total population, pooled results from 3 cities, n=3,802). Bars represent 95% CI, and significant associations are annotated with the corresponding odds ratio estimates.

**Figure S12**. **Sensitivity analyses: NO2, susceptible group.** Lag-specific odds ratios (red markers) and cumulative odds ratios (black squares) for COVID-19 per unit increase in air pollution exposure for NO2 in unconstrained lag models with different lag length (susceptible group, pooled results from 3 cities, n cases= 386). Bars represent 95% CI, and significant associations are annotated with the corresponding odds ratio estimates.

**Figure S13. Sensitivity analysis: NO2, excluding last COVID-19 wave.** Lag-specific odds ratios (red markers) and cumulative odds ratios (black squares) for COVID-19 per unit increase in NO2 exposure in unconstrained lag models with varying lag lengths (pooled results from 3 cities, excluding data from the final COVID-19 wave, n cases= 2,347). Bars represent 95% CI, and significant associations are annotated with the corresponding odds ratio estimates.
